# Genetic Testing in Patients with Hypertrophic Cardiomyopathy

**DOI:** 10.3390/ijms221910401

**Published:** 2021-09-27

**Authors:** Jiri Bonaventura, Eva Polakova, Veronika Vejtasova, Josef Veselka

**Affiliations:** Department of Cardiology, Motol University Hospital, 2nd Faculty of Medicine, Charles University, V Uvalu 84, 15006 Prague, Czech Republic; eva.polakova@fnmotol.cz (E.P.); veronika.vejtasova@fnmotol.cz (V.V.); josef.veselka@fnmotol.cz (J.V.)

**Keywords:** hypertrophic cardiomyopathy, genetics, molecular genetic testing, pathogenic mutations, variants of uncertain significance, next-generation sequencing

## Abstract

Hypertrophic cardiomyopathy (HCM) is a common inherited heart disease with an estimated prevalence of up to 1 in 200 individuals. In the majority of cases, HCM is considered a Mendelian disease, with mainly autosomal dominant inheritance. Most pathogenic variants are usually detected in genes for sarcomeric proteins. Nowadays, the genetic basis of HCM is believed to be rather complex. Thousands of mutations in more than 60 genes have been described in association with HCM. Nevertheless, screening large numbers of genes results in the identification of many genetic variants of uncertain significance and makes the interpretation of the results difficult. Patients lacking a pathogenic variant are now believed to have non-Mendelian HCM and probably have a better prognosis than patients with sarcomeric pathogenic mutations. Identifying the genetic basis of HCM creates remarkable opportunities to understand how the disease develops, and by extension, how to disrupt the disease progression in the future. The aim of this review is to discuss the brief history and recent advances in the genetics of HCM and the application of molecular genetic testing into common clinical practice.

## 1. Introduction

Hypertrophic cardiomyopathy (HCM) is an inherited cardiac disorder, defined by the presence of increased left ventricular (LV) wall thickness that is not solely explained by abnormal loading conditions [1,2]. In the majority of cases, HCM is considered a Mendelian disease with autosomal dominant inheritance, incomplete penetrance, and variable expressivity [3]. It is one of the most frequent inherited heart diseases with an estimated prevalence of up to 1 in 200 individuals and together with arrhythmogenic right ventricular cardiomyopathy (ARVC) among the most common cause of sudden cardiac death (SCD) in young athletes [4,5,6], who are often unaware of their underlying condition.

## 2. History of Finding the Cause of HCM

HCM was first described more than 60 years ago as asymmetrical myocardial hypertrophy with an increased risk of sudden cardiac death [7]. Although considered familial disease, the exact cause of HCM remained unknown for two subsequent decades.

Genetic studies in the 1980s and 1990s led to landmark discoveries that sarcomeric mutations cause both hypertrophic and dilated cardiomyopathies (DCM). In 1989, a mutation in the beta-myosin heavy chain (*MYH7*) gene was first identified as responsible for causing HCM [8,9]. During the next decade, numerous genes were reported to be associated with disease (Table 1) [10]. These eight sarcomeric genes (*ACTC1, MYBPC3, MYH7, MYL2, MYL3, TNNI3, TNNT2,* and *TPM1*) are commonly called core genes, with the most robust evidence to be causative of HCM (Table 1) [10,11]. This spectrum of sarcomeric genes has been gradually extended to non-sarcomeric genes encoding, for example, desmosomal proteins or ion channels [12,13]. However, a systematic evaluation of the investigation panels shows that the strongest evidence of causality remains in the eight core genes [11]. There is also strong evidence of causality in three genes—*PLN, FLNC* [11,14,15], and recently *ALPK3* [16] and moderate evidence of causality in five genes—*CSRP3, TNNC1, ACTN2*, *JPH2*, and *FHOD3* [17,18]. For the other genes, evidence is weak or almost non-existent [11,13,19]. Variants in genes encoding non-sarcomeric proteins account for a small percentage of patients with HCM. In light of recently published analyses, they seem to be the presumed causal genes at several genome-wide association study loci [17,20], and their role in cardiomyopathy genetics is gradually expanding. Currently published data demonstrate that common genetic variants and modifiable risk factors have important roles in the HCM phenotype [17].

Nowadays, more than 30 years after the publication of the first causal mutation in the MYH7 gene, thousands of mutations have been described and the numbers of identified HCM-associated genes are gradually increasing [21,22,23,24]. The Online Mendelian Inheritance in Man (OMIM) database currently lists 26 associated genes [25]. However, associations in at least 33 genes have already been reported [11] and 67 candidate genes are part of investigation panels at some expert sites [14].

It is clear, that genetic studies continue to demonstrate that HCM is predominantly a disease of the sarcomere, although the genetic basis of HCM is more diverse. Additionally, sarcomere mutations have been identified in association with other disorders of cardiac structure and function, apart from the above-mentioned DCM including restrictive cardiomyopathy and left ventricular non-compaction [26,27,28]. Moreover, recently published data suggest that shared genetic pathways contribute to HCM and DCM development with opposite directions of effect [20].

Genetic testing was initially possible only in research laboratories capable of performing linkage analysis and candidate gene sequencing in large, well-characterized families with obviously inherited diseases. The genetic and allelic heterogeneity of HCM makes molecular analysis by conventional methods time consuming and expensive [29,30]. Advances in contemporary DNA-sequencing methodology have made gene-based diagnosis increasingly feasible in routine clinical practice. Next-generation sequencing (NGS)-based genomic testing allows rapid analysis of a large number of genes or even a whole genome at similar cost and accuracy to conventional sequencing methods [30,31]. NGS is a high-throughput method that, in comparison with classical sequencing methods (Sanger), evaluates a large amount of genetic material quickly and is cheaper. NGS uses the principle of parallelization of the sequencing process, allowing the sequencing of thousands to millions of sequences simultaneously. In addition to classical examinations of genetic variability, mutation analysis of specific genes, and quantification of individual alleles, it is possible to examine the whole exome (WES) or to perform whole-genome sequencing (WGS).

Faster and more affordable genetic testing provides opportunities to improve diagnostic certainty when evaluating patients and families with relatively non-specific phenotypes of cardiac hypertrophy. With a molecular-level diagnosis, we can differentiate genetic sarcomeric HCM from phenocopies, such as hypertensive heart disease, athlete’s heart, and storage or metabolic disorders [32,33,34,35,36].

Nevertheless, screening large numbers of genes results in the identification of many genetic variants of uncertain significance (VUS) [30,31] and makes the interpretation of the results more difficult. The results of NGS produce a huge amount of output data with the subsequent need to sort and further analyze.

## 3. Identification of a Causative Mutation

For the clinical use of molecular genetic testing, the classification of the identified variants is essential. Due to a large amount of output data, a combined approach is currently used, based on the following rules:

- Frequency of variants in the control population, using international databases (e.g., 1000Genomes Project, Exome Sequencing Project, Exome Aggregation Consortium) [37,38,39]
- Published disease-associated variants (e.g., ClinVar, Human Gene Mutation Database) [40,41]
- In silico classification using software (e.g., Polyphen2, Sorting Intolerant From Tolerant) predicting the possible impact of the mutation on the structure and function of the final protein
- Mutations in the so-called evolutionarily highly conserved functional domains of the target protein
- Segregation analyses of genotype with phenotype in affected families (strong evidence)
- Functional studies on animal models or in vitro (expensive, complex)

In 2015, recommendations for the classification of genetic variants were published by the American College of Medical Genetics and Genomics (ACMG) and the Association for Molecular Pathology (AMP) [42], which is based on the above-listed principles. This classification divides the found variants into five following classes: (1) benign, (2) likely benign, (3) VUS—variant of unknown significance, (4) likely pathogenic (LP), and (5) pathogenic (P).

## 4. Genetic Screening

Genetic screening plays an important role in the management of patients with HCM and their relatives. The standard procedure is to obtain a detailed family history (at least three generations) and molecular genetic examination of the proband with a focus on at least all eight „core” sarcomeric genes associated with HCM (Table 1). If there is a clinical suspicion of a specific cause or HCM within the complex syndrome, then it is appropriate to expand the panel to other non-sarcomeric genes (Table 2).

In the case of a positive finding, molecular genetic testing of the first-degree relative for a specific gene and mutation already found in the proband is performed. If a pathogenic mutation is detected in a relative, a cascade examination of other relatives is possible (due to the predominant AD inheritance). Detailed family history and pedigree will help us to identify the probable hereditary cause of the disease and usually determine the type of heredity. Genetic analysis of post-mortem tissue samples with cascade screening of relatives is feasible [43]. The main clinical advantage is the situation where a specific causal mutation in the proband is not found in the first-degree relative. The relative can then be excluded from the dispensary, the probability of the disease is low, however, de novo mutations are possible. Therefore, we always warn patients about the need to seek a specialist in case of symptomatology. According to current recommendations, the examination of children is appropriate around the age of 6–10 [1,44]. The threshold was established based on pediatric studies, which showed a rare incidence of serious complications of HCM before the onset of puberty [45,46].

**Figure 1:**
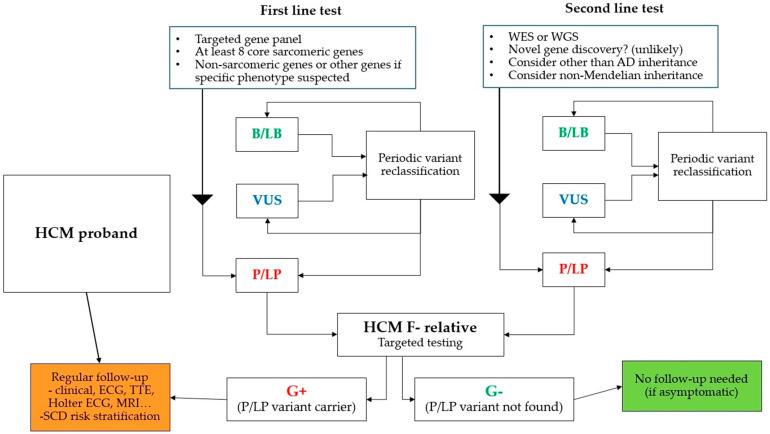
Cascade genetic testing. B/LB—benign or likely benign, P/LP—pathogenic or likely pathogenic, VUS—variant of unknown significance, WES—whole exome sequencing, WGS—whole genome sequencing, ECG—electrocardiography, MRI—magnetic resonance imaging.

If the molecular genetic examination of the proband is negative (no P/LP variant is found), we continue the established regular clinical monitoring of first-degree relatives. It includes clinical and echocardiographic examination, 12-lead ECG, Holter ECG monitoring (Figure 1). In selected patients (usually with insufficient echocardiographic window), cardiac magnetic resonance imaging (MRI) is performed. MRI can be useful in young patients with an early-onset screening of metabolic diseases [44,47] and its role in SCD risk stratification is increasing [44,48].

The opposite clinical situation is a clinically negative phenotype (F-) with the finding of P/LP mutation (genotype positive, G+). In contrast to DCM, where, for example, a mutation in the LMNA gene is associated with an unfavorable prognosis and is even part of the indication for ICD (implantable cardioverter-defibrillator) implantation according to ESC guidelines [49], the risk of SCD is generally low in individuals without expressed hypertrophy. Mutations in TNNT2 may be an exception, as suggested by some publications [50,51], but this is not strong evidence. It is not clear whether to make specific recommendations and propose restrictions, e.g., for professional athletes [21,52,53], based on a positive genotype without an expressed phenotype (G+/F-). It has been repeatedly reported that most G+/F- patients probably have a favorable prognosis [52,54]. However, due to age-related variable penetrance (55% to 30 years of age, up to 95% over 50 years of age [55], regular clinical monitoring of these individuals should be continued.

## 5. Diagnostic Yield of Molecular Genetic Testing

The diagnostic yield of genetic testing (detection of P/LP mutation) is variable and relatively low. The likelihood of detecting a pathogenic mutation is generally higher in younger patients and patients with a positive family history, where it can reach 50–60% [1,3]. In other patients, it is at most around 30–40% [3] and with the introduction of stricter ACMG classification criteria, the yield is even lower [42,47,49]. The high sensitivity of genetic testing with the advent of NGS methods was thus gradually replaced by higher specificity related to the application of complex classification criteria. It appears that genetic data and the classification of pathogenic mutations from past decades will need to be revised in the context of current knowledge [42].

The relatively low yield of genetic testing required the emergence of clinical scoring systems to predict its positivity. The best known and probably the most accurate is the Mayo HCM Genotype Predictor score (Mayo Score), which is based on basic clinical and echocardiographic parameters and was first published in 2014 [56]. Predictors of the yield of genetic testing include age < 45 years, left ventricular wall thickness 20 mm and more, family history of HCM, family history of SCD, and the so-called reverse (catenoid) shape of the interventricular septum. A negative predictor is the presence of arterial hypertension (Table 3).

In the original Mayo cohort, the overall yield of genetic testing was 34% when examining nine genes for sarcomeric proteins (*ACTC1, MYBPC3, MYH7, MYL2, MYL3, TNNC1, TNNI3, TNNT2,* and *TPM1*) [56]. With the development of NGS, the gene panels began to differ significantly in the number and type of individual genes, depending on the local availability and financial capabilities of sites.

The aim of our Czech study published in 2019 [57] was to assess the yield of genetic testing using the classification according to the ACMG/AMP guidelines [42] and validate the previously established and published Mayo Score in the national HCM cohort using the stringent ACMG/AMP classification criteria. In our study, we evaluated up to 229 cardiac condition-related genes. All of our three testing gene panels included the eight core sarcomeric genes. Despite the vast number of included genes, the overall yield of genetic testing was only 21% [57].

Our relatively low yield and low frequency of mutations in certain genes may account for more stringent criteria for calling a variant disease-associated over time. In the past, studies might have allowed a variant to be called disease-associated solely based on its absence in 50–100 reference alleles in healthy controls [58,59]. In the original Mayo cohort, case-derived variants that were absent in more than 8400 healthy controls or seen with a frequency of <0.01% in controls and significantly overrepresented in cases versus controls were included as G+ [56]. Since that time, data from projects such as 1000 Genomes [37], or ExAC [38] have demonstrated how many variants previously deemed to be pathogenic are present at population frequencies incompatibly high with Mendelian dominant disease causation. Two years after the publication of the original Mayo Score paper, the same authors used a new cohort of HCM patients to validate the original genotype predictor score [60]. In the validation study, variants classified as likely pathogenic, possible, or probably pathogenic, or VUS were considered G+ [60]. If we used a similar approach in our study, the yield of genetic testing would be 42% [57].

In all the above-mentioned analyses [56,57,60] the yield of genetic testing was consistently predicted with Mayo Score values. Its use appears to be rational in clinical practice, where financial constraints are evident. Due to the time-consuming nature of molecular genetic testing (usually months), a large number of negative results and overuse of the NGS method can be avoided by careful selection of patients.

## 6. Genotype and Phenotype Correlation

The genetic heterogeneity of HCM is enormous [21,22,23] and the relationship of a single mutation to a specific typical phenotype has mostly not been satisfactorily explained so far. So-called private mutations are very common in families and therefore genotype-phenotype correlation is not possible on a larger group of patients. Variable penetrance is evident especially in the *MYBPC3* gene, where it can lead to a completely different phenotype within individual families [61]. In the *MYBPC3* gene, so-called founder mutations are a common finding, which is highly conserved within various geographically or culturally isolated populations. Penetration is typically postponed until later in age [3,61,62].

With the availability of molecular genetic testing using high-throughput methods, we can investigate an increasing number of genes. However, it does not lead to a higher diagnostic yield, as described in the previous chapter. Final variant classification requires more correlated genotypes with phenotypes and segregation analyses [30,31]. Publicly available databases, as ClinVar [40], for reporting mutations and their relationship to the phenotype are of the most importance.

Due to this genetic heterogeneity, the data for genotype-phenotype relationships are still insufficient. Some studies show that the finding of a pathogenic mutation (G+) in HCM patients worsens cardiovascular mortality, increases the risk of stroke and progression of NYHA class symptomatology compared to genotype negative (G−) patients [50]. According to recent work by other authors, G+ patients are at higher risk of SCD and have higher overall and cardiovascular mortality [63]. However, most of these published data on the relationship between specific mutated genes and the severity of the phenotype is on a relatively small number of patients from a single center.

The association of a mutation in the MYH7 gene with early disease onset and risk of cardiovascular events has been described for pediatric patients [64]. Published data from the Portuguese HCM patient registry suggest an association of a mutation in the same gene with LV systolic dysfunction in adults as well [65]. In this relatively large registry (528 molecularly genetically examined patients), the finding of any pathogenic mutation (G +) was also associated with a higher risk of SCD [65].

Data from probably the largest multicenter registry of genotyped patients with HCM—SHaRe (Sarcomeric Human Cardiomyopathy Registry)—show that the predictor of adverse prognosis is, in addition to younger age at the diagnosis of HCM, also P/LP mutation in one of the typical sarcomeric genes [66]. According to the SHaRe registry, the finding of a P/LP mutation is associated with a 2-fold increased risk of a combined endpoint (all-cause death, heart failure, malignant arrhythmias, atrial fibrillation). The finding of VUS in sarcomeric genes increases this risk approximately 1.5-fold. The risk of developing severe LV systolic dysfunction (6-fold) and the need for mechanical cardiac support or heart transplantation (4-fold) is more likely when P/LP mutations are found [66]. Interestingly, the register also contains data of patients who have at least two P/ P mutations (they are compound or double heterozygotes) and make 2.8% of the group of patients with a positive genotype. The incidence of the combined endpoint was comparable (HR 1.06) to patients with a single P/LP mutation, but these patients tended to develop more frequent severe LV systolic dysfunction and the need for mechanical cardiac support or heart transplantation (HR 7.5). The comparative analysis of P/LP mutations in the two most common genes—MYH7 and MYBPC3—is also unique. Consistent with the above-cited work, a 1.7-fold higher risk of combined endpoint was found for mutations in the MYH7 gene versus mutations in the MYBPC3 gene [66].

An important factor in the interpretation of the findings is incomplete penetrance and variable expressivity typical for HCM [61]. This suggests that factors other than mutations at the sarcomere protein level play a role in the long-term clinical course of the disease. Other genetic and epigenetic factors, as well as environmental modifications, probably play an important function that we cannot yet satisfactorily characterize [67]. Thus, when we talk about HCM as a disease with AD Mendelian inheritance, it is a significant (and probably wrong) simplification of the true disease nature [68,69,70]. Genetic modifiers usually include DNA methylation and acetylation, and the importance of miRNA has recently been discussed [24]. It is possible that newly discovered variants of non-sarcomeric genes, e.g., for ion channels, desmosomes, or titin, are not the primary cause of HCM, but the mentioned modifiers [12,17,71,72,73].

In clinical practice, the coincidence of HCM with arterial hypertension is common (due to the high prevalence of both diseases). The hemodynamic situation in arterial hypertension necessarily modifies the HCM phenotype. Despite respecting the exclusion criteria for the diagnosis of HCM [1,44], we also encounter patients with severe aortic stenosis, concomitant diagnosis of arterial hypertension, and HCM. Exercise (beware of the athlete’s heart) and other comorbidities such as diabetes mellitus, obesity, and chronic renal failure also lead to modification of the resulting HCM phenotype. Obesity and arterial hypertension are associated with a larger volume of LV myocardium [74]. Obesity has been proposed as an important HCM phenotype modifier and associated with worse outcomes [75,76,77]. Diastolic blood pressure appears to be a substantial risk factor for the development of sarcomere-negative HCM according to recent data [17]. The relationship between arterial hypertension and HCM is obviously even more complicated [78].

The importance of sex differences in HCM patients is more and more discussed. HCM has shown a male predominance, with men comprising approximately 60% of most published HCM [79]. Consistently with different penetrance [80,81] women are usually older at the time of diagnosis [82,83]. Women are also known to have a higher prevalence of the obstructive phenotype, worse diastolic function, and more severe heart failure symptoms at presentation [82,84]. Increased overall mortality [82] and worse outcomes after septal reduction therapy [79,85,86] were also described.

From the above information, it is clear that it is often impossible to distinguish the real cause or the main modifier of LV hypertrophy in clinical practice. Currently, the intensively researched disease is the so-called senile (wild-type transthyretin) amyloidosis, which is also expected to have a relatively high prevalence in the general population [87]. It could be effectively treated [88] if the correct diagnosis of this HCM phenocopy is made on time.

In the field of all the above comorbidities, determining the true etiology of LV hypertrophy can be very complicated [67].

## 7. Future

Identifying the genetic basis of HCM creates remarkable opportunities to understand how the disease develops, and by extension, how to disrupt disease progression [89]. With the development of novel therapies [90,91,92] to target these pathways to delay or prevent full clinical expression, genetic discoveries can change medical practice. In mice, some mutant alleles may be effectively silenced [93]. Nevertheless, mutation-silencing therapies are likely to be ineffective for LV hypertrophy regression and would have to be administered very early in life to prevent hypertrophy development [94]. Recent advances in precise genome-editing techniques and their successful applications in animal models have provided an option for correcting human germline mutations. In particular, CRISPR-Cas9 is a useful tool for recognizing specific genomic sequences and inducing double-strand breaks [95]. The correction of the heterozygous *MYBPC3* mutation in human preimplantation embryos with precise CRISPR-Cas9-based targeting accuracy and high homology-directed repair efficiency by activating an endogenous, germline-specific DNA repair response was recently reported [96]. From an ethical point of view, the possibility of editing the human germline is at least controversial. Especially in HCM, taking into account incomplete penetrance and variable expressivity, there will certainly be a complicated path from preimplantation or another prenatal diagnosis, to the most aggressive decision—pregnancy interruption. The finding of a pathogenic mutation in an otherwise healthy embryo or fetus does not necessarily mean the development of the disease in the lifetime, and even in the case of HCM development, the clinical course is variable and the prognosis in most patients is almost comparable to the general population [97].

Many questions must be answered to translate genetic findings to enhance the care of patients. With current knowledge, we fail to identify mutations in sarcomere genes in more than half of HCM patients [15,57]. We seek a more complete understanding of the burden of genetic disease in “genotype-negative” patients and the identification of other disease-causing genes. It is clear that a large proportion of individuals with a clinical diagnosis of HCM but without sarcomere gene mutations may exhibit a distinct disease process that has a more complex, non-Mendelian inheritance pattern [98,99,100].

Although comprehensive genetic testing, such as WES or WGS, will identify new genes implicated in cardiomyopathy, a substantially higher number of VUS will also be generated, potentially increasing overall ambiguity. A more sophisticated understanding of human genetic variation and more robust approaches to assess the pathogenicity of sequence variants are needed to complement the massive amount of information returned from comprehensive genotyping. With the vast amount of newly discovered VUS, the multidisciplinary team is at risk of burden with difficult-to-interpret variants that can psychologically stress or even cause iatrogenic harm to the subjects or their families. Respecting this caveat, and the cost-effectiveness policy, prediction of diagnostic yield of genetic testing using the simple scoring systems [56,57,101] or more complex methods [102,103,104] could become routinely used.

We need to gain a better understanding of the great phenotypic diversity of sarcomere mutations and the modulation of gene expression throughout a patient’s lifetime. We should try to fully characterize genetic, epigenetic, and environmental modifiers and explain the diverse clinical manifestations and outcomes. Larger cohorts that will include genotyping and longitudinal clinical phenotypes could provide further insights. Given the enormous heterogeneity of these conditions, multicenter collaborations will be essential for success.

## 8. Take-Home Message

HCM is characterized by significant phenotypic and genotypic variability. Genetic testing by current methods, including NGS, does not detect any significant mutations in more than half of the patients. With modern methods comes the possibility of examining a large number of genes, including WES and WGS. The volume of these data, especially the interpretation of VUS, requires the close cooperation of a cardiologist, molecular biologist, and clinical geneticist. Factors influencing the development of HCM in genotype-negative patients (G−/F+) and asymptomatic mutation carriers (G+/F−) have not yet been satisfactorily elucidated. Extensive genetic studies in large cohorts of related patients would be needed to fully understand the genotype–phenotype relationship, the effect of genetic background, and comorbidities on disease development and course. Despite the relatively high prevalence of the disease, this can only be achieved through the international cooperation of large centers, the standardization of detailed genetic testing, and the interdisciplinary cooperation of cardiologists, clinical geneticists, and molecular biologists.

## Figures and Tables

**Table 1:** Main sarcomeric genes associated with HCM.

Gene	Protein	Year of Discovery	Frequency (%) *	Inheritance	Most Common Pathogenic Variant
	**Thick filament**				
MYH7	Beta-myosin heavy chain	1989	20–30	AD	c.1988G>A
MYL2	Regulatory myosin light chain	1998	2–4	AD	c.173G>A
MYL3	Essential myosin light chain	1996	1–2	AD	c.281G>A
	**Thin filament**				
TNNT2	Cardiac troponin T	1993	10	AD	c.236T>A
TNNI3	Cardiac troponin I	1997	7	AD	c.433C>T
TPM1	Alpha tropomyosin	1993	<1	AD	c.574G>A
ACTC1	Alpha cardiac actin	1999	<1	AD	c.301G>A
	**Intermediate filament**				
MYBPC3	Myosin-binding protein C	1993	30–40	AD	c.1504C>T

AD—autosomal dominant, * Indicates relative frequency in HCM population.

**Table 2:** Non-sarcomeric genes associated with HCM.

Gene	Protein	Phenotype	Prevalence *	Inheritance	Frequency (%) **
PRKAG2	Protein kinase, AMP-activated, gamma 2 subunit	Wolff–Parkinson–White syndrome	1/4000	AD	0.2–1.0
LAMP2	Protein kinase, AMP-activated, gamma 2 subunit	Danon disease	1/100,000	X	0.1–0.2
GLA	Galactosidase, alpha	Fabry disease	1/40,000	X	0.5–1.0
FHL1	Four and a half LIM domains 1	Emery–Dreifuss myopathy	1/100,000	X	0.1–0.5
TTR	Transthyretin	Amyloidosis ***	1/100,000	AD	0.8–5
GAA	Glucosidase, alpha	Pompe disease	1/40,000	AR	0.01–0.1
PTPN11	Protein tyrosine phosphatase, non-receptor type 11	Noonan syndrome LEOPARD	1/2000	AD	1–5
FXN	Frataxin	Friedreich ataxia	1/20,000	AR	0.05–0.2

AR—autosomal recessive, X-X linked, * Indicates prevalence in the general population, ** Indicates relative frequency among HCM cases, may differ from the expected prevalence in the general population due to the selection bias of HCM genotyped cohorts, *** hereditary, not wild-type (senile).

**Table 3:** Mayo HCM Genotype Predictor Score [56].

Clinical Variable	Points
Age < 45 years	1
Left ventricular wall thickness > 20 mm	1
Family history of HCM	1
Family history of sudden cardiac death	1
Reverse septal shape	1
Arterial hypertension	−1

## Data Availability

Not applicable.
